# First person – Sandra Muñoz-Braceras

**DOI:** 10.1242/dmm.039230

**Published:** 2019-02-22

## Abstract

First Person is a series of interviews with the first authors of a selection of papers published in Disease Models & Mechanisms, helping early-career researchers promote themselves alongside their papers. Sandra Muñoz-Braceras is first author on ‘ VPS13A, a closely associated mitochondrial protein, is required for efficient lysosomal degradation’, published in DMM. Sandra conducted the research described in this article while a postdoctoral fellow in Ricardo Escalante's lab at Instituto de Investigaciones Biomédicas Alberto Sols, CSIC/UAM, Madrid, Spain. She is now a postdoctoral fellow in the lab of Rosa Puertollano at the Cell Biology and Physiology Center, National Heart, Lung, and Blood Institute, National Institutes of Health, Bethesda, USA, investigating lysosome contribution to the maintenance of cellular homeostasis.


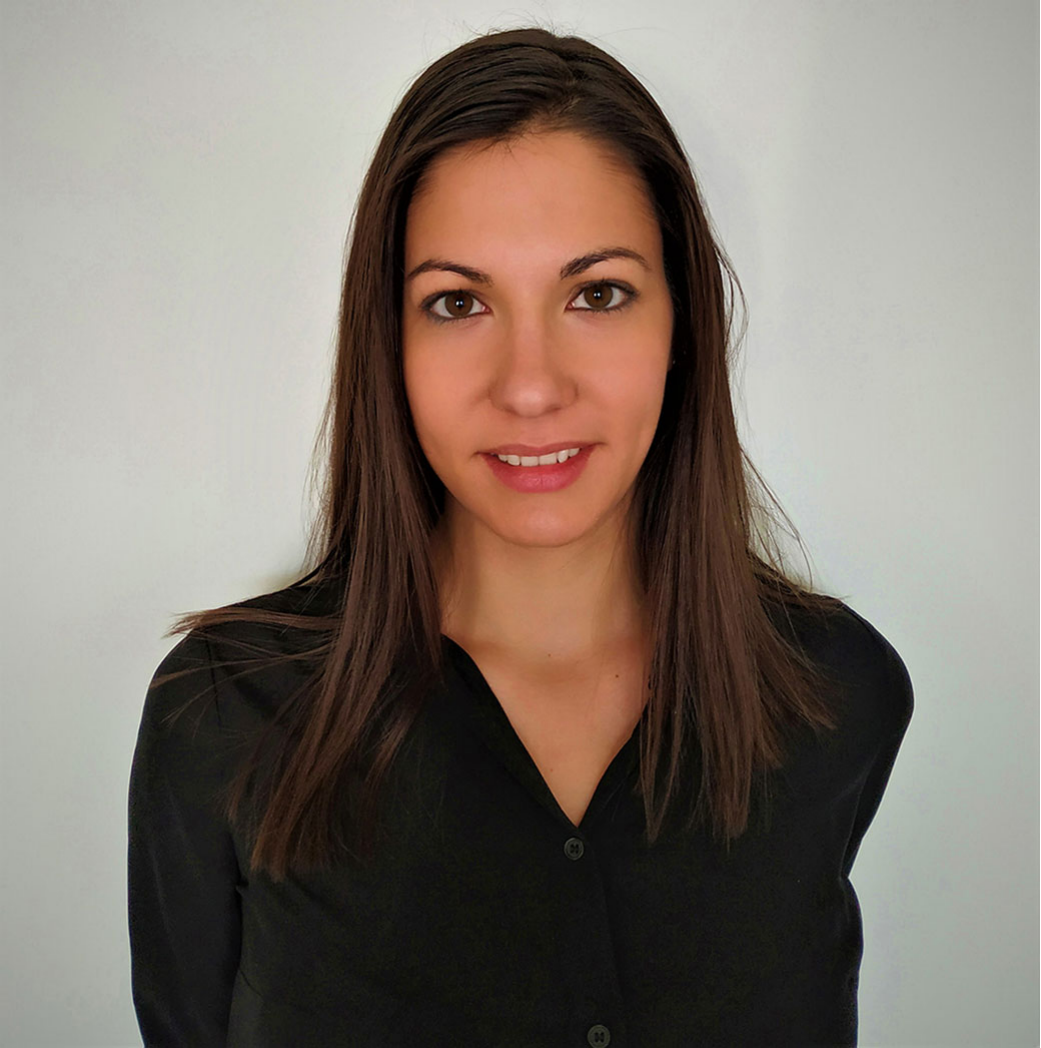


**Sandra Muñoz-Braceras**

**How would you explain the main findings of your paper to non-scientific family and friends?**

Our research is focused on VPS13A, a protein that is absent in people suffering from a rare neurodegenerative disease known as chorea-acanthocytosis (ChAc). In order to know what happens at a cellular level during the disease, we treated cultured cells to deplete VPS13A and discovered the defective degradation of material within a type of vesicle, called lysosomes, in these cells. Lysosomal degradation is essential to keep our cells healthy and we think that its dysfunction can be related to the progression of ChAc. We do not know yet why the absence of VPS13A leads to diminished lysosomal degradation, but our observations suggest that this effect might be related to a defective communication between lysosomes and mitochondria, a different cellular structure with which most VPS13A is associated.

“[…] our research provides a reasonable basis to consider lysosomal-related pathways as a potential target for the treatment of ChAc, for which there are no known therapies to stop or slow down the progression of the disease.”

**What are the potential implications of these results for your field of research?**

From a molecular perspective, I think that our findings will promote further research focused on the roles of VPS13A related to the physical contact between mitochondria and lysosomes, and on the functional connection between these organelles. From a more translational point of view, I believe that our research provides a reasonable basis to consider lysosomal-related pathways as a potential target for the treatment of ChAc, for which there are no known therapies to stop or slow down the progression of the disease.

**What are the main advantages and drawbacks of the model system you have used as it relates to the disease you are investigating?**

We have mostly used human cells from an immortal cell line, but it is worthwhile mentioning that a finding using *Dictyostelium discoideum*, an amoeba able to form multicellular structures, was the starting point of our research. These models are easily manipulated in the laboratory, but divergence during evolution (in the case of dictyostelids) or cell-type specificity in addition to potential changes during the process of immortalization (in the case of human cell lines) can lead to differences in the field of study. However, concerning research on ChAc, these model systems have proven useful for creating a hypothesis about the etiology and pathophysiology of the disease. In fact, our previous research using a mutant of a Vps13 protein in *D. discoideum* inspired us to study autophagy, a lysosomal-related pathway, which we subsequently found was partially impaired in immortal human cells depleted of VPS13A, and was later described by other colleagues to be defective in red blood cells from ChAc patients.

**What has surprised you the most while conducting your research?**

The subcellular localization of VPS13A. The observation of most VPS13A signal being associated with mitochondria was totally unexpected considering that our results indicated that VPS13A is necessary for the proper function of lysosomes, and that it interacts with a protein characterized to be on endolysosomal membranes.

**Figure:**
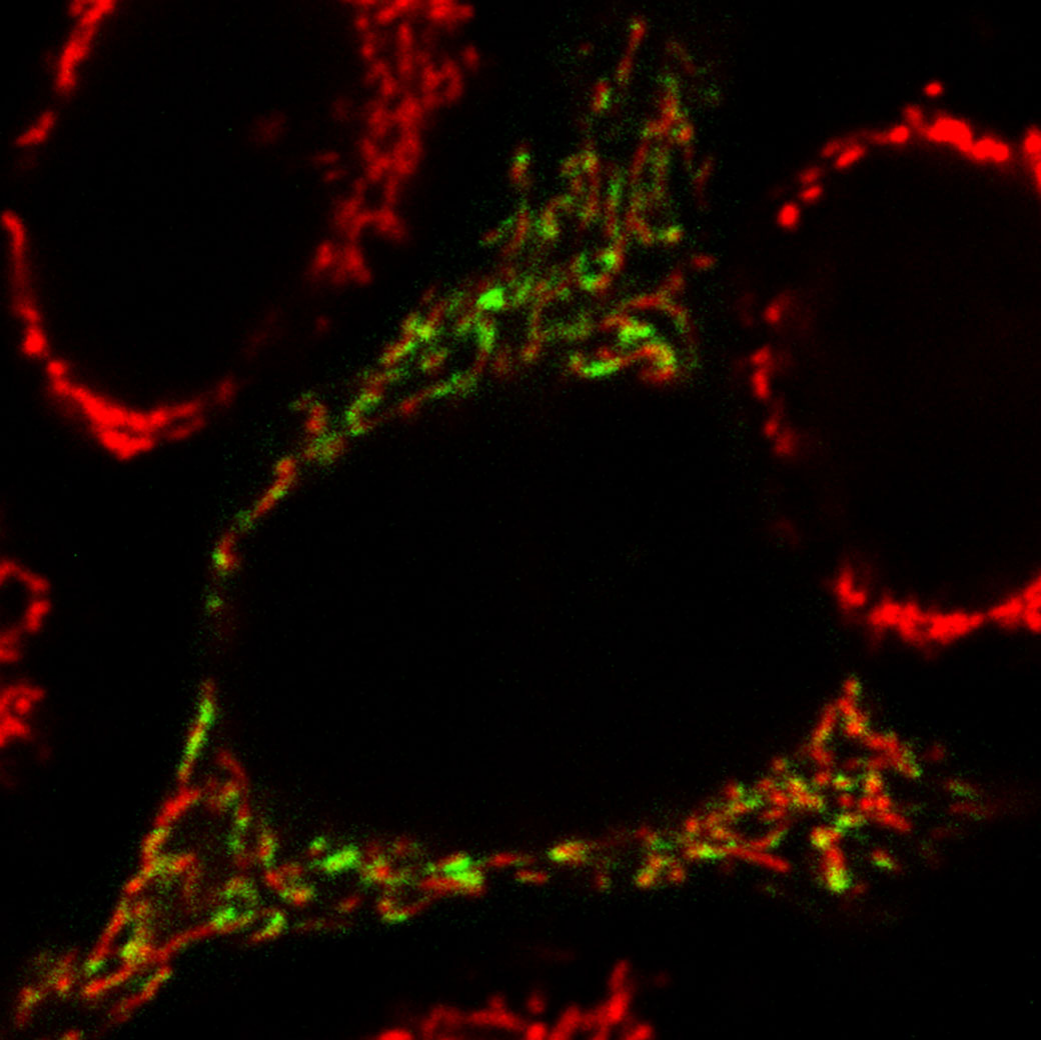
**VPS13A (green) is closely associated with mitochondria (red).**

**Describe what you think is the most significant challenge impacting your research at this time and how will this be addressed over the next 10 years?**

In a broad sense, I think that VPS13 proteins, in general, are a challenge. They are very large proteins and they probably have multiple functions. Focusing on VPS13A and the lysosomal-related role that we have proposed, I consider that the first question to be answered is if lysosomal degradation is actually impaired in the affected neurons of ChAc patients and, if so, the contribution of this potential defect to the etiology and progression of the disease. While this might be very difficult to assess in patients, it could be addressed in neurons generated from ChAc fibroblasts, in which abnormalities in lysosomes and mitochondria have been very recently described. If the functional evaluation of lysosomes in ChAc cells validates our results, understanding the molecular mechanisms by which the absence of VPS13A results in decreased lysosomal function will be necessary to know how to restore the lysosomal-related pathways. This still requires more research to be performed using simple model systems, such as those that we have used during this work.

“[…] if early-career scientists had more opportunities to continue doing research, their professional lives would improve; in addition, with their experience, science in general would improve.”

**What changes do you think could improve the professional lives of early-career scientists?**

In my opinion (I am only speaking personally), more funding could substantially improve the lives of scientists. My own conviction is that it would not only make cutting-edge technologies more accessible to scientists and favor them to achieve their greatest potential, but also allow many more early-career scientists to simply continue their career. More experience should lead to more opportunities, but it seems to me that it is quite the opposite in the scientific career, where I dare say that there are great difficulties to find positions to pursue a career in research after a postdoctoral temporary period; mainly because, from my point of view, many laboratories cannot afford to hire a staff scientist (at least in Spain, where I come from). I believe that if early-career scientists had more opportunities to continue doing research, their professional lives would improve; in addition, with their experience, science in general would improve. For those starting their career in research and realizing how frustrating it can be, I consider that a positive mindset, enthusiastic and supporting mentors, and inspiring collaborations can make a remarkable positive difference in their lives.

**What's next for you?**

I have just started a new postdoctoral position and I will continue doing research for some years, but I do not discard moving out of the bench in the future. I like the idea of exploring other sectors where I can both learn totally new things and apply the scientific expertise and other skills gained in the laboratory.
